# Report of July Meeting

**Published:** 1861-08

**Authors:** Charles G. Smith

**Affiliations:** Secretary; Chicago


### CHICAGO MEDICAL SOCIETY.

The Society met as usual. The Sanitary Committee was appointed by the President, as follows: Dr. Holmes for the North Side, Dr. Davis for the South Side, and Dr. H. W. Jones for the West Side. Dr. Davis made a verbal report for his division, and Dr. Holmes also. Dr. Holmes reported eight cases of diptheria in his practice, during the last four weeks, three of which were fatal.

Dr. Davis reported a case of poisoning by arsenic. The poison was sent from some drug store in this city, instead of cream of tartar.—[See paper appended.]

Dr. Dickinson, of St. Louis, was introduced to the Society, and brought to their notice a new anæsthetic, discovered during the manufacture of kerosene, and, for the present, named *kerosolene.* [A paper from Dr. Dickinson will be found in another portion of this number, embodying all that is as yet known of this article.] On motion of Dr. C. G. Smith, the kerosolene was referred to a committee, with a request to examine and report on its qualities at the next meeting. The Chair named as the committee, Dr. Amerman, Dr. Orrin Smith, Dr. Wickersham.

Dr. Wickersham related a case of partial anæsthesia, produced by inhaling the vapor of the oil of turpentine.

*Asthma*, the proposed subject, was then discussed by Dr. Amerman, Dr. C. G. Smith, Dr. Waite, Dr. Orrin Smith, and Dr. Wickersham. Dr. Amerman showed an interesting specimen of aneurism of the aorta, in which a large secondary sac had been found, which finally communicated to the trachea, causing death. Dr. C. G. Smith exhibited a specimen of fractured skull.

On motion of Dr. Wickersham, it was voted, that reports of cases shall come before the regular discussion, in the order of business.

The following was chosen as a subject for discussion for the next meeting: "*The influence of Iodide of Potassium on the system.*"

Dr. Reilly was chosen as the essayist.

CHARLES G. SMITH, *Secretary.*

CHICAGO, 19th July, 1861.

